# Alveolar Macrophages Infected with Ames or Sterne Strain of *Bacillus anthracis* Elicit Differential Molecular Expression Patterns

**DOI:** 10.1371/journal.pone.0087201

**Published:** 2014-02-07

**Authors:** Felicia D. Langel, Chih-Yuan Chiang, Douglas Lane, Tara Kenny, Jenifer F. Ojeda, Yang Zhong, Jianwei Che, Yingyao Zhou, Wilson Ribot, Krishna P. Kota, Sina Bavari, Rekha G. Panchal

**Affiliations:** 1 Uniformed Services University of the Health Sciences, Bethesda, Maryland, United States of America; 2 Molecular and Translational Sciences Division, U.S. Army Medical Research Institute of Infectious Diseases, Fort Detrick, Maryland, United States of America; 3 SAIC-Frederick, Inc., Frederick National Laboratory for Cancer Research, Frederick, Maryland, United States of America; 4 Genomics Institute of the Novartis Research Foundation, San Diego, California, United States of America; 5 Bacteriology Division, U.S. Army Medical Research Institute of Infectious Diseases, Fort Detrick, Maryland, United States of America; 6 Perkin Elmer, Waltham, Massachusetts, United States of America; The University of Texas-Houston Medical School, United States of America

## Abstract

Alveolar macrophages (AMs) phagocytose *Bacillus anthracis* following inhalation and induce the production of pro-inflammatory cytokines and chemokines to mediate the activation of innate immunity. Ames, the virulent strain of *B. anthracis*, contains two plasmids that encode the antiphagocytic poly-γ-d-glutamic acid capsule and the lethal toxin. The attenuated Sterne strain of *B. anthracis*, which lacks the plasmid encoding capsule, is widely adapted as a vaccine strain. Although differences in the outcome of infection with the two strains may have originated from the presence or absence of an anti-phagocytic capsule, the disease pathogenesis following infection will be manifested via the host responses, which is not well understood. To gain understanding of the host responses at cellular level, a microarray analysis was performed using primary rhesus macaque AMs infected with either Ames or Sterne spores. Notably, 528 human orthologs were identified to be differentially expressed in AMs infected with either strain of the *B. anthracis.* Meta-analyses revealed genes differentially expressed in response to *B. anthracis* infection were also induced upon infections with multiple pathogens such as *Francisella Novicida* or *Staphylococcus aureus*. This suggests the existence of a common molecular signature in response to pathogen infections. Importantly, the microarray and protein expression data for certain cytokines, chemokines and host factors provide further insights on how cellular processes such as innate immune sensing pathways, anti-apoptosis versus apoptosis may be differentially modulated in response to the virulent or vaccine strain of *B. anthracis*. The reported differences may account for the marked difference in pathogenicity between these two strains.

## Introduction

*B. anthracis,* the causative agent of anthrax, is a Gram-positive spore-forming bacterium. *B. anthracis* can infect its host through respiratory, cutaneous or gastrointestinal routes. Inhalation anthrax is the most lethal form of the disease associated with high fatality rate and occurs when endospores enter the lungs during respiration. Ames is a virulent strain of *B. anthracis* which contains three main virulence factors encoded on two plasmids, pXO1 and pXO2. The pXO1 plasmid encodes anthrax edema and lethal toxins (ET and LT, respectively), which are composed of three proteins, the edema factor (EF), the protective antigen (PA), and the lethal factor (LF). The pXO2 plasmid encodes genes that are responsible for the synthesis of a layer of poly-γ-d-glutamic acid (PGA) capsule outside of the bacterial cell wall [1]. The capsule is relatively non-immunogenic and allows *B. anthracis* to escape immune-surveillance of the host [2]. Furthermore, this capsule may protect *B. anthracis* from phagocytosis. Under appropriate conditions, *B. anthracis* spores germinate into vegetative bacilli, disrupt phagocytes and penetrate into the circulatory system where high levels of the toxin are secreted. The Sterne strain of *B.* anthracis is an attenuated strain that does not carry the pXO2 plasmid (pXO1^+^, pXO2^−^) and thus is not encapsulated, making it more susceptible to phagocytosis.

Host innate immune system expresses Toll-like receptors (TLRs) that recognize components of *B. anthracis*. For example, anthrolysin O (ALO), a cholesterol-dependent cytolysin (CDC) secreted by *B. anthracis*, is a potent agonist of TLR4. Exposing bone marrow derived macrophages (BMDMs) to ALO results in robust up-regulation of proinflammatory cytokine gene expression [3]. Following engagement of ligands to TLR4, adaptor proteins MyD88 and TRIF are recruited to the receptor, resulting in the activation of transcription factors NF-κB and interferon regulatory factor-3 (IRF-3), respectively [4]. Mitogen-activated protein kinase (MAPK) pathway is also employed upon TLR activation to orchestrate the expression of pro-inflammatory and interferon stimulated genes (ISGs) [5]–[7]. However, *B. anthracis* has evolved ways to impair these host responses and thereby allowing growth of bacteria and establishment of infection. For instance, LF is a zinc-dependent bacterial protease that cleaves the amino terminus of mitogen-activated protein kinase kinases 1 and 2 (MEK1 and MEK2) and this cleavage inactivates MEK1 and inhibits the MAPK signal transduction pathway [8]–[11]. Also, EF is a highly active bacterial adenylyl cyclase that elevates intracellular concentrations of cyclic AMP and de-regulates cellular gene transcriptions.

The Ames strain gained public attention in association with the 2001 anthrax attacks. Because of its virulence, the Ames strain is used as a benchmark for testing the effectiveness of any new vaccines that are under development [12]. Immunization using the live, attenuated Sterne strain is able to stimulate a protective immune response and is effective as a vaccine against domesticated animals for many decades worldwide. It has also been used to vaccinate humans in Eastern European countries [13], [14]. Although the mechanism of how *B. anthracis* toxins impairs host immune systems has been well characterized, cellular responses to Ames versus Sterne spore infections has not been fully investigated in primary cells. The objective of this study is to profile host transcriptional responses after exposing rhesus macaque AMs to either the virulent Ames or attenuated vaccine Sterne strains of *B. anthracis*. We present evidence that primary AMs exhibit differential transcriptional gene expression profiles following infection with Ames or Sterne spores. A total of 913 rhesus macaque genes, which are matched to 528 human orthologs, were identified to be differentially expressed using microarray analysis. These 528 genes are hierarchically clustered into 4 subsets based on their expression patterns over the time course study. Microarray gene expression was further validated by quantifying mRNA or protein expression levels of several cytokines and chemokines. For example, AMs infected with Sterne spores preferentially induced the production of TNF-α, CCL5 and CCL3. Furthermore, expression patterns of cyclooxygenase-2 (COX-2) and prostaglandin E_2_ (PGE_2_), which are important mediators of inflammation upon pathogen infection, were also investigated. AMs infected with Ames spores exhibit delayed expression of COX2 gene which in turn correlates with a delay in production of PGE_2_ expression.

## Materials and Methods

### Cells and Reagents

The lungs of rhesus macaque (*Macaca mulatta*) were purchased from Lonza (Walkersville, MD). Primary AMs from rhesus macaque were harvested by flushing lungs with cold PBS/EDTA/gentamicin solution. Cells were pelleted by centrifugation at 240×g for 5 min, washed once in PBS/EDTA/gentamicin solution, and centrifuged again. To purify AMs, cells were allowed to adhere for 2 h in 5% CO_2_ at 37°C, after which non-adherent cells were removed and adherent cells were further incubated overnight in complete Dulbecco’s Modified Eagle Medium (cDMEM) that contains high-glucose, 7.5% FBS, 1% L-Glutamine, and 1% non-essential amino acids (NEAA). Cell viability was determined by trypan blue staining. A representative cell sample was evaluated for purity by staining with May-Grünwald-Giemsa stain of air-dried cytospin smears [11]. During *B. anthracis* infection studies, all the plates were briefly centrifuged to promote the adherence of the spores to AMs. AMs infected with *B. anthracis* for greater than 120 mins were washed and further incubated for indicated time points with medium containing gentamicin (50 µg/ml) to kill extracellular bugs.

Ames strain (pXO1^+^, pXO2^+^) and Sterne strain (pXO1^+^, pXO2^−^) of *B. anthracis* were prepared as previously described [15]. The Green fluorescent protein (GFP) expressing Sterne spores [11], [16] and GFP expressing Ames spores (pXO1^+^, pXO2^+^) (unpublished data) were kindly provided by Wilson Ribot (USAMRIID) and were used only for the imaging studies.

The anti-MEK1 N-terminus (NT), p44/p42, phospho-p44/p42, and phospho-p38 antibodies were obtained from Millipore. Anti-p38 antibody was obtained from Cell signaling Technology. Anti-GAPDH antibody was obtained from Life Technology. Latex beads used in western blot experiments were purchased from Sigma Aldrich.

### Imaging B. anthracis Spores in Infected AMs

For fluorescent imaging studies, purified AMs (40.000 cells/well) plated in a 96 well plate (Greiner bio-one µ clear poly-D-Lysine coated 96 well plate: #655946) were infected with GFP-Ames or GFP-Sterne spores at a multiplicity of infection (MOI) of 10. Infected macrophages were fixed at indicated times (1 h, 2 h and 6 h), stained with Hoechst 33342 (Life Technology #H3570) and Cell Mask Deep Red (Life Technology #H32721). Images were acquired by Leica SP5-STED confocal microscope with 63X water objective. Internalization of the spores was verified by acquiring images in Z series.

For Transmission Electron Microscopy (TEM) studies AMs infected with Ames spores at MOI of 10 were fixed at the indicated time with 4% formaldehyde/1% glutaraldehyde for seven days at room temperature, rinsed 2–3 times in a modified Millonig’s buffer (0.1M PBS, 0.5% dextrose; pH 7.3), and treated for 1–2 h with 1% osmium tetroxide in modified Millonig’s buffer just prior to Transmission Electron Microscopy (JEOL 1011) visualization. TEM samples were stained *en bloc* with 0.5% uranyl acetate in ethanol, dehydrated in graded ethanol and propylene oxide, and embedded in Poly/Bed 812 resin (Polysciences, Inc.). Ultra-thin sections were placed on 200-mesh nickel grids and stained with 5% uranyl acetate and 0.2% lead citrate.

### Quantifying the Viability of AMs

Cell viability of *B. anthracis* infected macrophages was performed as described previously [17]. Briefly, adherent AMs or murine J774A.1 macrophages were uninfected or infected with Sterne spores at an MOI of 10. Four hours post infection; cells were resuspended in cDMEM with 1% penicillin/streptomycin to kill the bacteria. Cell viability was measured by adding 50 µl of 1∶500 diluted SYTOX green dye (Life Technology). The cells were further incubated for 15 min, washed in cDMEM, and resuspended in 1% formaldehyde. Cells were immediately analyzed by flow cytometry.

### Bacterial Viability

Purified AMs (∼250,000 cells/well) seeded in 24 well-plate were infected with Sterne or Ames strains of *B. anthracis* at an MOI of 10. After 0, 30, 60, 120, 240 and 360 minutes post-infection, cells were washed three times with PBS and lyzed using PBS containing 0.1% TX-100. Each sample was diluted appropriately and then plated onto drug free blood agar plates to determine the number of CFU present in the sample. CFU is the number of colonies on the plate multiplied by the dilution factor.

### Immunoblot Analysis

Purified AMs (∼2 x 10^6^ cells/well of a 6-well plate), were either left untreated/uninfected or treated with LPS (100 ng/ml), latex beads (LB) or infected with Ames or Sterne spores at MOI of 10. After 30 mins or 6 h, cells were washed with PBS and transferred into tubes following scraping. AMs were pelleted and lysed in the lysis buffer (50 mM Tris-HCl [pH 7.4], 150 mM NaCl, 2 mM EDTA, 25 mM β-glycerophosphate, 1% Triton X-100, 10 mM NaF, 1 mM Na_3_VO_4_, protease inhibitor cocktail, phosphatase inhibitor cocktails I and II). Thirty microgram of total protein per sample was resolved by SDS-PAGE. Gels were transferred onto the nitrocellulose membrane and probed with protein-specific antibodies. Proteins were visualized by enhanced chemiluminescence (ECL) (Thermo Scientific). Densitometry of the western blot was computed by Image J.

### Quantifying Cytokine and Chemokine Production

Purified AMs seeded in a 6 well-plate (∼2 x 10^6^ cells/well) from three independent donors were stimulated with 100 ng/ml LPS or infected with Ames or Sterne spores at an MOI of 10. Supernatants were collected at 90 min, 4 h and 18 h post -treatment. For the 18 h time point, gentamicin (50 µg/ml) was added 4 h post infection to kill the extracellular bacteria. Pro-inflammatory cytokine levels were quantified by using Cytometric Bead Array (CBA) Human Inflammatory Cytokine Kit (BD Biosciences). CCL3 and CCL5 levels were quantified by using Human 25-Plex Panel (Life Technology). PGE_2_ level was quantified by using Prostaglandin E_2_ Human ELISA Kit (Life Technology). Secreted PGE_2_ was quantified in the supernatant. Data were analyzed using FCAP Array software, Bio-Plex Manager 4.1.1 software or Microsoft Office Excel.

### Microarray Analyses

Purified AMs from five independent rhesus macaques (∼2×10^6^ cells/well of a 6 well-plate) were infected with Ames or Sterne spores at an MOI of 10. At indicated time points of 30 min, 90 min, 120 min, 240 min and 360 min, cells were washed three times with PBS to remove the extracellular bacteria and Trizol solution (Life Technology) was directly added to each well to lyze the cells and total cellular RNA was isolated according to the manufacturer’s protocol. mRNA was purified and reverse transcribed using high Capacity cDNA Reverse Transcription Kits (Life Technology) and the quality and concentration of RNA were verified. mRNA was reverse transcribed into cDNA and hybridized to the NHP genome array (Affymetrix, Inc.), which consists of over 39,000 genes. All microarray data were processed with both gcRMA algorithm and MAS5 algorithm. MAS5 algorithm marked each probe set as Present (“P”), Marginal (“M”), or Absent (“A”) within a hybridization. If a probe set was marked as “A” in more than three out of five host replicates, it was considered as absent in the given biological condition. If a probe set was absent across all time points, it was removed from further analysis. A paired t-test was applied between the two treatment groups of each time point, respectively, where expression data from the same host were paired. Those probe sets with P-value ≤0.05 and fold change more than cutoffs were considered as differentially expressed genes and were retained. The fold change cutoff thresholds for each time point were determined by the average two standard deviations calculated from linear regression of two treatment data at each corresponding time point, which is always more than two fold (i.e., absolute difference between their average log2 gcRMA expression levels is at least 1.0). Only probe sets that could be mapped to rhesus macaque Entrez Gene IDs according to the latest Affymetrix annotation file (release 33) were retained. When multiple probe sets mapped into the same gene, the probe set with the largest statistically significant fold change value in any conditions was retained as the representative probe set of the gene. According to the latest NCBI Homologene database, probe sets were also mapped to human orthologs via their rhesus macaque gene IDs. The process described above resulted in 913 unique differentially expressed rhesus macaque genes and 528 unique differentially expressed human genes.

### Real-Time Polymerase Chain Reaction (Real-time PCR)

AMs (∼2×10^6^ cells/well of a 6 well-plate) from three donors were stimulated with 100 ng/ml LPS or infected with Ames or Sterne spores. At indicated time points, cells were washed with PBS and lyzed directly in the well using Trizol solution. Total cellular mRNA was isolated using TRIzol solution (Life Technology) and genomic DNA was eliminated by DNase I treatment. RNA samples were reverse transcribed using RT^2^ First Strand Kit (SABiosciences Corporation). PCR products were detected using the Power SYBR Green PCR Master Mix (Life Technology). For each experimental sample, triplicate reactions were prepared for each gene of interest and the signal is normalized to a housekeeping gene to account for any differences in cDNA recovery between samples. Primers used for real-time PCR assay were purchased from SABioscience Corporation. The catalogue numbers for GAPDH and COX-2 are PPQ00249A and PPQ16024A, respectively.

### Bioinformatics Analyses

Enriched Gene Ontology groups were identified for the hit lists using a standard accumulative hyper-geometric P-value analysis. GO groups with P-value ≤0.01 were retained. Comparative microarray analyses were achieved by utilizing NextBio [18]. Transcription factor binding sites in gene promoters were predicted by SABiosciences Text Mining Application and the UCSC Genome Browser.

## Results and Discussion

### Phagocytosis and Subsequent Killing of B. Anthracis Spores by Alveolar Macrophages

**Figure 1:**
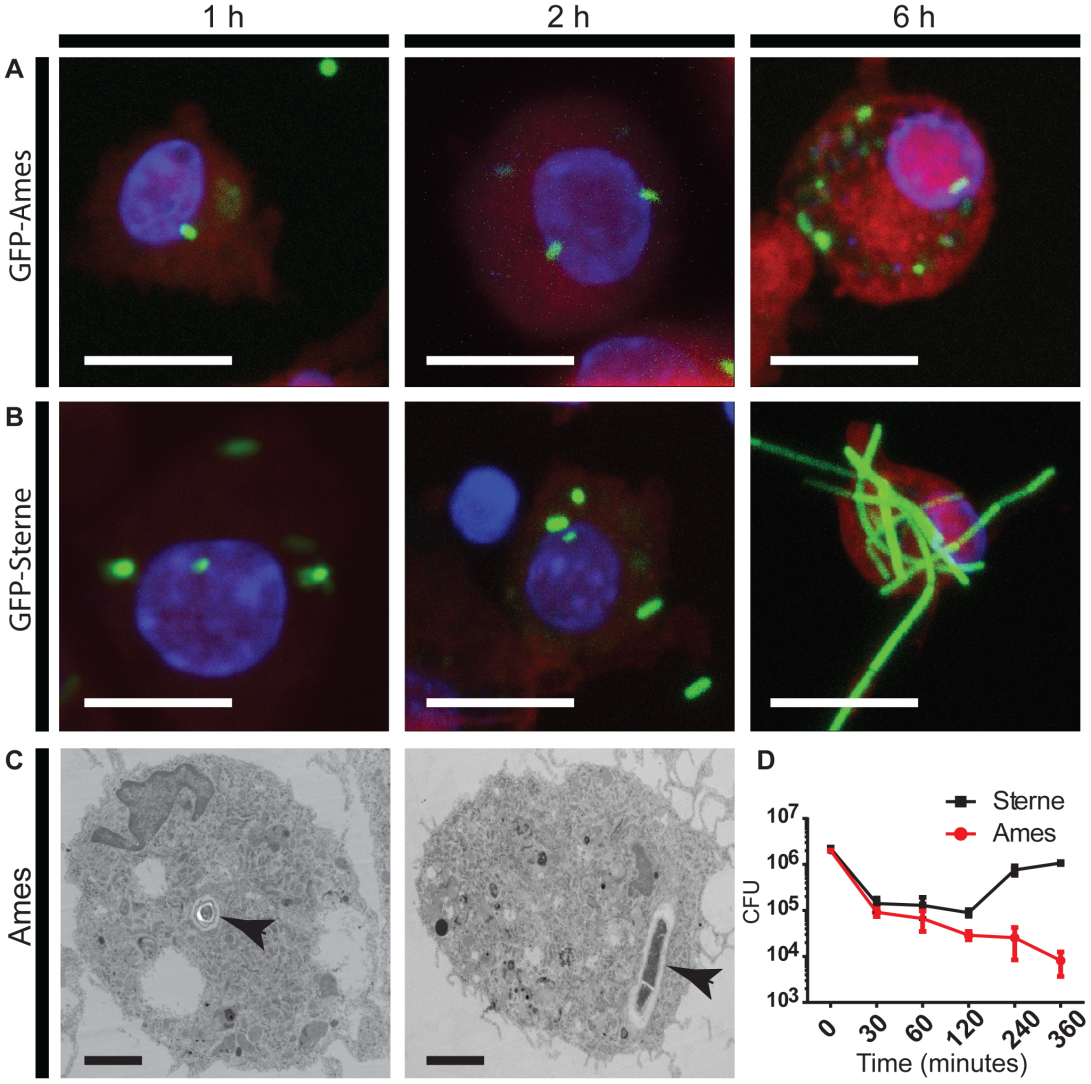
AMs phagocytose and kill *B. anthracis* spores. (A & B) AMs were infected with GFP-Ames or GFP-Sterne spores for indicated time points and imaged using confocal microscopy. Phagocytosed spores and vegetative bacilli appear bright green. Scale bar = 10 µm. Images presented are at maximum projection. (C) AMs were infected for indicated time points with unlabeled Ames spores and imaged using transmission electron microscopy. The internalized spores are indicated with the arrows. Scale bar = 2 µm. (D) AMs were infected with Ames or Sterne spores for different time points and bacterial viability was determined using the colony forming assay. The data represents averages from three independent NHP AMs ± SEM.

To investigate host responses to *B. anthracis* infection, we first confirmed that Ames and Sterne strains of *B. anthracis* spores were taken up by AMs and were able to germinate, thus mimicking the actual host infection. Phagocytosis of GFP-Ames (Figure 1A) or GFP-Sterne (Figure 1B) spores by AMs was visualized by confocal microscopy, while the unlabeled phagocytosed Ames spores were detected using transmission electron microscopy (TEM) (Figure 1C). All three strains of *B. anthracis* were phagocytosed by AMs following infection. However, in comparison with the Ames strain, engulfment of Sterne spores and subsequent outgrowth into vegetative bacilli was observed as early as 2 h post-infection and distinct long chains of vegetative bacteria were visible by 6 h post-infection. This data suggests that AMs have the ability to phagocytose both Ames and Sterne spores and those bacteria that survive are able to germinate and replicate within the cytoplasm. The observed differences in the vegetative growth kinetics between Sterne and Ames *B. anthracis* may be attributed to the presence of the anti-phagocytic capsule in the Ames *B. anthracis*.

We next investigated if the AM could efficiently kill the phagocytosed *B. anthracis*. AMs were infected with Ames or Sterne *B. anthracis* spores and bacterial viability were determined at various time points post-infection using the standard colony forming assay. As shown in Figure 1D, there is a rapid decline in viable bacteria within 30 minutes post-infection. The bacterial viability continued to decline with a slower rate for up to 6 h post–infection for Ames, while the Sterne strain returned to the initial levels at 6 h post-infection. These results suggest that the AMs are functionally active as they are able to kill phagocytosed bacteria. The Sterne strain replicated more robustly by 6 h post-infection, and thus further supporting the vegetative growth observed by confocal microscopy (Figure 1B). On the contrary, the continuous decline in the viability of Ames strains up to 6 h post-infection could be due anti-phagocytic capsule resulting in a shift in the growth kinetics.

### B. Anthracis Infection Modulates MAPK Signaling

**Figure 2:**
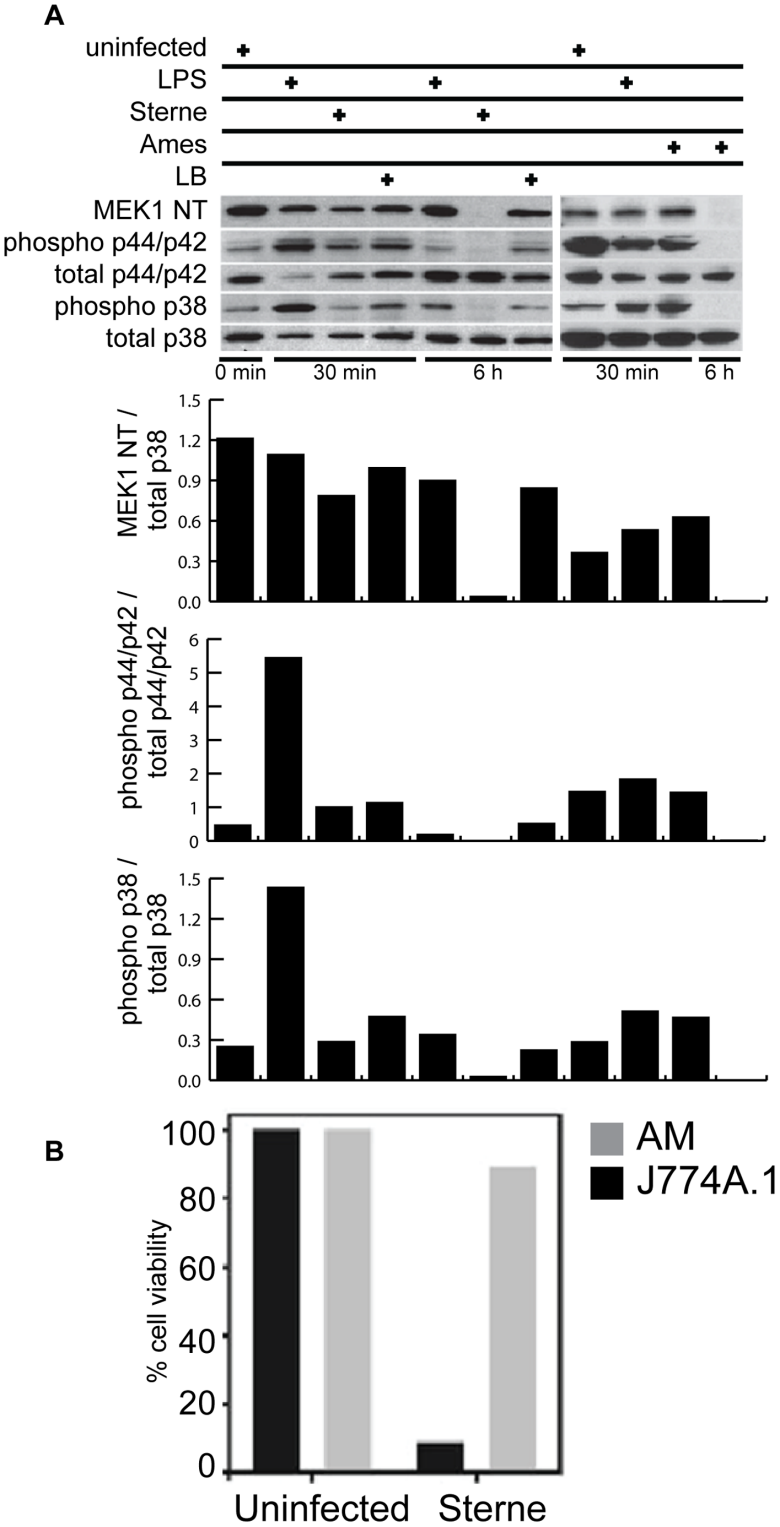
*B. anthracis* mediated MEK1 degradation does not impair AM survival. (A) AMs were either left uninfected, incubated with 100 ng/ml of *E. coli* lipopolysaccharide (LPS), Latex beads (LB) or infected with *B. anthracis* Sterne or Ames spores at an MOI of 10. Thirty minutes or 6 h post-infection, whole cell lysates were prepared and immunoblotted with antibodies against MEK1 NT, phospho-p44/p42, total p44/p42, phospho-p38 and total p38. Data shown is representative of n = 3 experiments. The bottom panel is a bar graph of the densitometric scan and is depicted as a ratio of the pixel intensities for the two indicated proteins. (B) AMs or J774A.1 macrophages were either not infected or infected with Sterne spores at an MOI of 10. Four h post infection, cell viability of AMs was determined by the uptake of the membrane impermeable SYTOX green dye using flow cytometry analysis.

We have previously reported that AMs treated with externally added purified LT were susceptible to LT-induced MEK1 cleavage, but did not affect AM viability [11]. Infection of AMs with Ames and Sterne spores (both LT producing *B. anthracis*) showed complete MEK1 cleavage at 6 h post infection (Figure 2A, top panel), a pattern similar to that observed with externally added LT [11]. Cleavage of MEK1 did not affect the integrity of p44/p42 and p38 but instead impaired its ability to phosphorylate p44/p42 and p38 and as confirmed by densitometry scans of the western blots (Figure 2A, bottom panel). As a negative control, AMs incubated with latex beads (LB) showed normal MEK1 expression. To determine if MEK1 cleavage would result in cell death, AMs were infected with Sterne spores and cell viability was quantified by the uptake of membrane impermeable Sytox green dye [19]. A greater than 90% cell survival rate was observed in Sterne infected AMs while less than 10% viability was observed in J774A.1 macrophages, an LT-susceptible cell line (Figure 2B). These results, together with our previously published studies with externally added *B. anthracis* LT [11], demonstrate that both externally added LT as well as LT produced following *B. anthracis* infection causes the AMs to become susceptible to MEK1 cleavage and resistant to the killing effects by Sterne *B. anthracis*.

### AMs Infected with Sterne or Ames Elicit Differential Pro-inflammatory Responses

**Figure 3:**
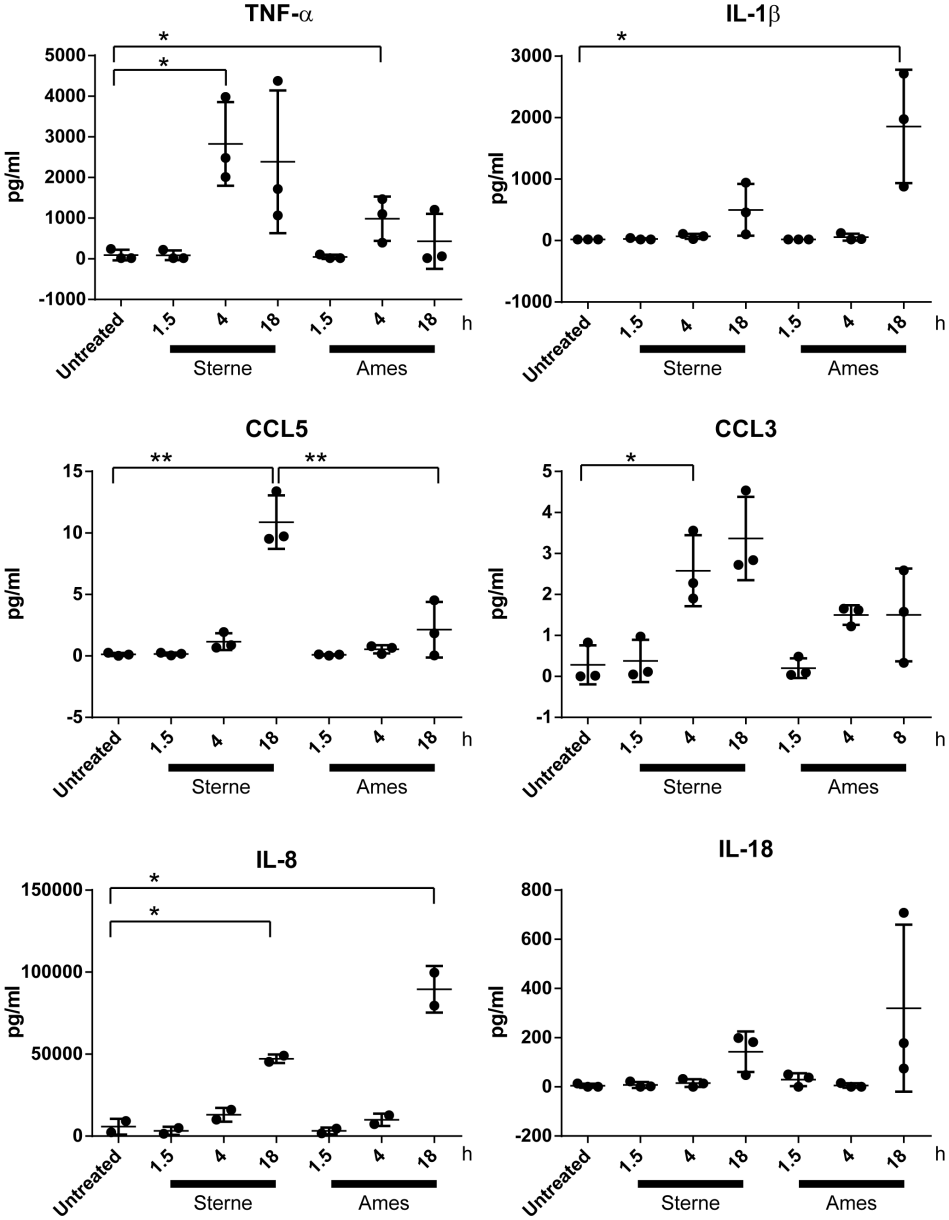
AMs infected with Ames or Sterne spores show differential pro-inflammatory cytokine/chemokine protein expression patterns. AMs were infected with either Ames or Sterne spores at an MOI of 10. Supernatants were collected at indicated time points and cytokine/chemokines levels were quantified. The experiment was performed at least three times and data for three rhesus macaque donors are shown. Scatter plots are presented as mean ± Standard Deviation. Experiments for Figure 3 and Figure S1 were performed concurrently and shared the same controls. P-value is calculated using a paired Student’s t-test. *represents p-value <0.05 and **represents p-value <0.01.

As effectors of innate immunity in the lungs, AMs elicit a robust cytokine response when encountering pathogen infections. To examine the effects *B. anthracis* spore infection on cytokine and chemokine secretion, AMs were infected with Ames or Sterne spores for 90 min, 4 h, or 18 h and supernatants collected. Cytokines and chemokines such as TNF-α, IL-1β, CCL5, CCL3, IL-8 and IL-18 were induced following infection (Figure 3). As compared to uninfected controls, AM infected with Sterne or Ames showed significantly increased levels of TNF-α (p<0.05) at 4 h and IL-8 (p<0.05) at 18 h post-infection, while IL-1β was significantly induced by Ames only at 18 h post-infection. The chemokines CCL3 (p<0.05) and CCL5 (p<0.01) were significantly induced by Sterne only at 4 h and 18 h post-infection, respectively. Comparison of the induced cytokine/chemokine levels between Ames and Sterne infection revealed significantly increased levels of CCL5 (p<0.01) only in Sterne infected AM at 18 hrs post-infection. A biased cytokine response in favor of TNF-α and CCL5 secretion in Sterne-infected AMs and IL-1β secretion in Ames-infected AMs suggests the triggering of fundamentally different signal cascade by the host immune responses.

To achieve the maximum anti-microbial effect, multiple receptors of the innate immune system can detect the presence of a pathogen. Both TLRs and NOD like receptors (NLRs) have been reported to detect *B. anthracis* components. For example, *B. anthracis* cell wall components and PA are sensed by TLR2/6 hetero-dimers and anthrolysin O is a potent agonist of TLR4 [3], [20]. NOD2, an intracellular sensor of bacterial LF, also plays a key role in the *B. anthracis*-induced IL-1β production [21]. Two distinct signaling cascades are required for the production of mature IL-1β. The first signal event is responsible for the production of IL-1β precursor which is predominantly mediated by the activation of NF-κB. Secondly, in response to *B. anthracis* infection, IL-1β precursor is cleaved by an inflammasome that is composed of NOD2, NALP-1 and caspase-1 [21]. Our data suggests that NF-κB signaling pathway is activated after infection with both Ames and Sterne spores. This is measured by the production of TNF-α and IL-8, which are NF-κB dependent genes. The fact that IL-1β secretion was up-regulated in AMs infected for 18 h with Ames but not with Sterne spores suggests that inflammasome is preferentially activated in response to infections with Ames spores.

### Differential Gene Patterns between Sterne and Ames Infected AMs

**Figure 4:**
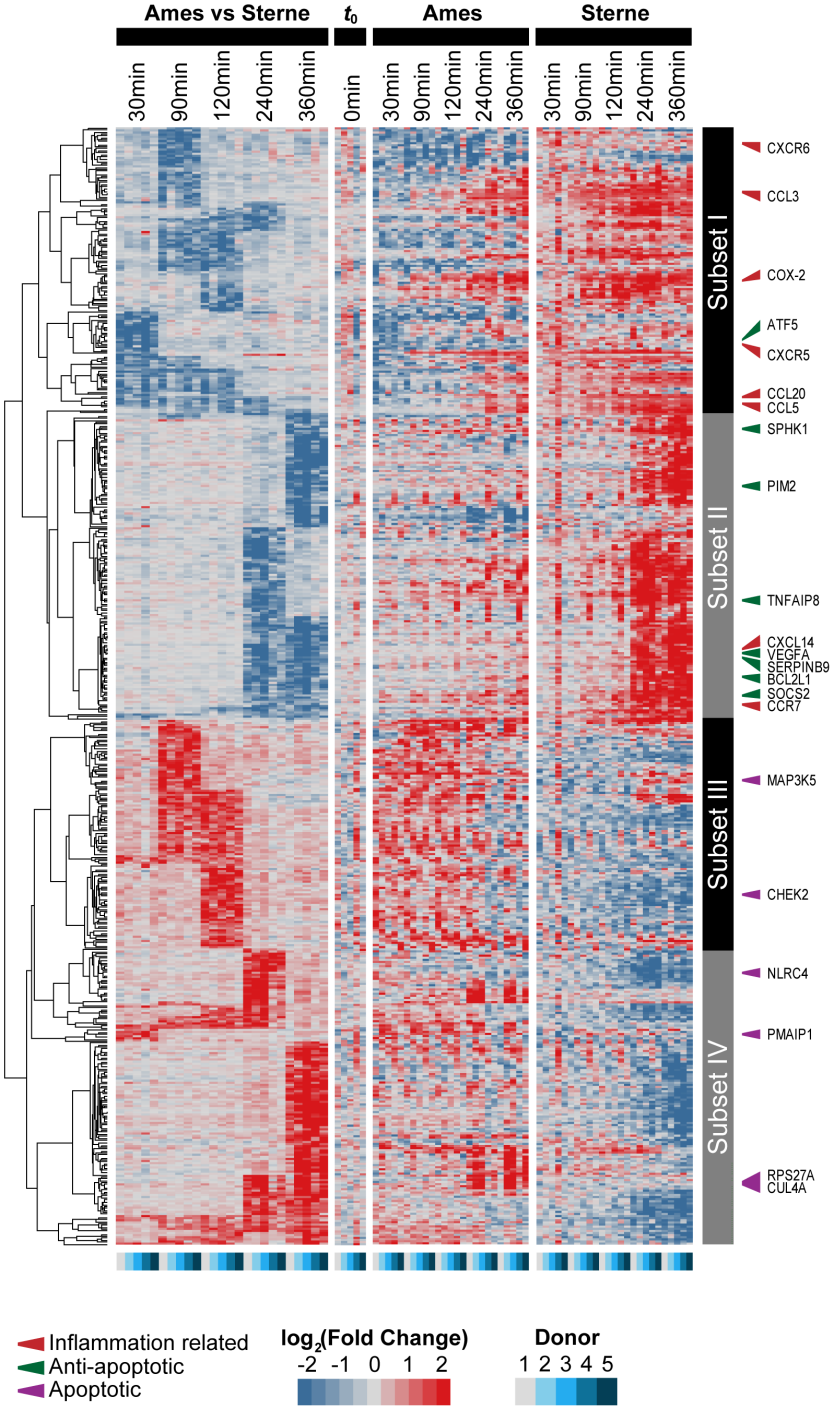
Microarray analyses identified 528 human orthologs which were differentially expressed between Ames and Sterne infected AMs. AMs obtained from five rhesus macaque donors were infected with Ames or Sterne spores at an MOI of 10 for indicated time points. Total mRNAs were purified and hybridized to rhesus macaque cDNA microarrays. “Ames vs Sterne” bar depicts the ratio of gene expressions (in logarithmic scale) between Ames-infected AMs to Sterne-infected counterparts. “Ames” and “Sterne” bar depict fold change of a gene expression (in logarithmic scale) by normalizing Ames or Sterne treated AMs with the 0 h time point. The level of fold changes are colored coded, where red stands for high values (>1) and blue for low fold changes (<1). To highlight statistically significant changes for “Ames vs. Sterne” for the ease of visualization, we decreased the contrast by three folds for the portion of the heat map, where differential expression is not significant.

The observed differences in cytokine responses for AMs infected with Ames or Sterne spores led us to investigate the global gene expression pattern of AMs in response to *B. anthracis* infection. Rhesus macaque non-human primate (NHP) Affymetrix gene chip arrays were used to compare the gene expression patterns in AMs infected with either wild type Ames or Sterne spores at 30 min, 90 min, 2 h, 4 h, or 6 h time points. Several genes (CCL20, NR4A2 and NR4A3) that were observed to be up-regulated in this study had previously been reported in a microarray study conducted in human AMs infected with the Sterne spores and served as quality controls [22]. A total of 913 unique differentially expressed rhesus macaque genes were identified in the study, which correspond to 528 human orthologs. Genes that show differential expression levels were hierarchically clustered into 4 major subsets based on the 1) kinetics of the gene expression pattern, and 2) their similarities or differences in the expression pattern in Ames vs. Sterne infected AMs (Figure 4 & Table S1). Subsets I and II represent genes whose expressions are significantly higher in Sterne infected AMs at early and delayed time points, respectively. Subsets III and IV represent genes whose expressions are significantly higher in Ames infected AMs at early and delayed time points, respectively.

**Figure 5:**
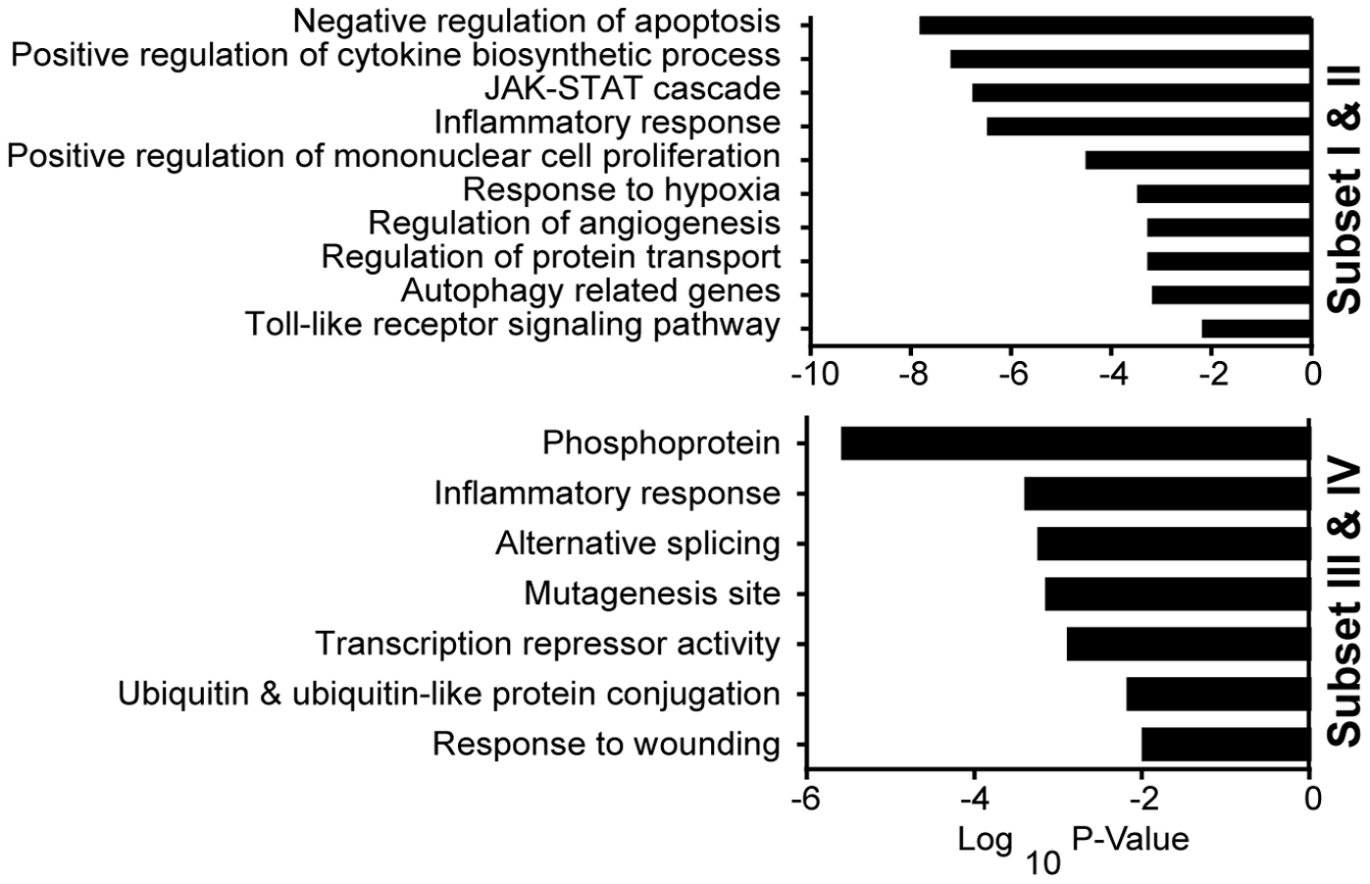
Gene ontology (GO) analysis. Statistically significant overrepresentation of selected functional classes and protein families based upon gene ontology (GO) analysis.

Gene Ontology (GO) analyses revealed the enrichment of pathways and cellular processes associated with inflammatory response, regulation of T cell proliferation and TLR signaling pathways. Pathways that are not well characterized in response to *B. anthracis* spore infection were also identified in the study. For example, genes that are involved in negative regulation of apoptosis, autophagy and response to hypoxia were identified in subsets I & II; whereas pathways involved in alternative splicing and ubiquitin protein conjugation were identified in subsets III and IV (Figure 5 & Table S2). Furthermore, meta-analyses suggested that genes differentially expressed in response to *B. anthracis* spores infection were also induced upon infections with multiple other pathogens such as *Francisella novicida*, *Staphylococcus aureus*, Ebola virus and severe acute respiratory syndrome coronavirus (SARS), etc. Interestingly, the most significant overlaps were between *B. anthracis* and other bacterial infections (*F. novicida*, *S. aureus*) (Table 1). This suggests the existence of common molecular signatures in response to bacterial infections.

**Table 1:** Overlap between the genes identified from current study and studies referenced.

Pathogens	# of overlappinggenes	Cells used in the study	Selected overlapping genes	P-value ofthe overlap	PMID ID orGEO ID
**Genes preferentially upregulated in Sterne infected AMs**					
Francisellanovicida	166	Human monocytes	IL6; PTGS2; TFPI2; CCL20; IL23A; IL1A; PTGS2; IL1F9; TNF; TFPI2	4.50E-39	18698339
Staphylococcalaureus	157	Human monocyte-derived macrophages	STAT4; ITGB8; WNT5A; OSM; SERPINB9; VEGFA; SLC25A37; SLC1A2; EHD1; TSLP; GFPT2; SIK1	4.70E-48	19381294
EbolaVirus	39	NHP PBMC	ZNRF2; GPR183; BCL3; UST; MEF2D; PDE5A; TNFAIP8; STARD13; ZNF331; BCAT1	4.90E-08	17725815
MeaslesVirus	25	Human CD14+ monocytes	HIVEP2; STAT4; MAMLD1; CREM; MSC; ADORA2A; RAPGEF2; TNRC6B; UPP1; NR4A1	3.20E-06	16492729
Humancytomegalovirus	83	Human Monocyte	IL6; IL12B; TNFAIP6; CCL20; PTGS2; TNF; ITGB8; ADORA2A; TRAF1	8.30E-17	18566437/20173022
SARScoronavirus	156	Human Lung Tissue	SLAMF7; CCL3; SOCS1; CLEC4E; IL12B; IL1A;MARCKSL1; BCAT1; IL1F9; AREG	1.50E-16	Series GSE33266
NewcastleDisease Virus	195	Human monocyte-derived dendritic cells	CCL5; SLAMF7; NCF1; MASTL; IRAK2; SLAMF7;TNFSF15; TRIM5; HIVEP2; CREM	9.00E-16	20164420
HumanRhinovirus	175	Bronchial epithelial cells	B3GALT6; EHD1; THBD; IRAK2; ATF5; SPHK1; BEST1;TBC1D10B; THBS1; ARHGDIA	9.40E-07	19710636
**Genes preferentially upregulated in Ames infected AMs**					
EbolaVirus	67	NHP PBMC	CACNB4; DUSP6; TCF4; NSL1; ELMO1; OPN3; UXS1; HHEX; IPCEF1; SGK1; SLC39A10; SLC39A10	1.10E-08	17725815
SARScoronavirus	96	Human Lung Tissue	CXCL10; NXT2; CCL7; EHF; SEPSECS; SLC8A1; C3orf58; DPYD; RBM8A; MINPP1; SLC39A10; SLC39A10	2.30E-07	Series GSE33266

Explanation of the column headings in table are as follows: Pathogens: name of the pathogen used in the referenced microarray studies; # of overlapping genes: the number of genes that are overlapping between the current study and the referenced studies; Cells used in the study: the type of cells used in the referenced microarray studies; Selected Overlapping Genes: selected gene symbols that are overlapping between the two studies; P-value: the P-value of the overlap; PMID or GEO ID: the reference of the published microarray studies.

Two of the well characterized chemokines CCL5 and CCL3 are crucial for immune responses towards infection and inflammation [23]. Our microarray analyses showed that these two chemokines were differentially expressed after Ames and Sterne infections (Figures 4 and S1, Table S1). Consistent with the microarray analyses was the observed rapid induction of CCL3 protein at 4 h post infection but at lower levels in Ames compared to Sterne infection (Figure 3). In addition, similar differential expression patterns for CCL5 were observed between Sterne and Ames infection both at the gene and protein levels after 4 h or 18 h post-infection respectively. Taken together, these results suggest that AMs infected with Ames or Sterne display differential cytokine and chemokine gene and protein expression patterns. Furthermore, a time-dependent expression of certain chemokines and cytokines may govern the outcome of the immune response upon *B. anthracis* infection.

### B. anthracis Infected AMs Demonstrate a Time-dependent Kinetic Difference in the Induction of COX-2 and PGE_2_

**Figure 6:**
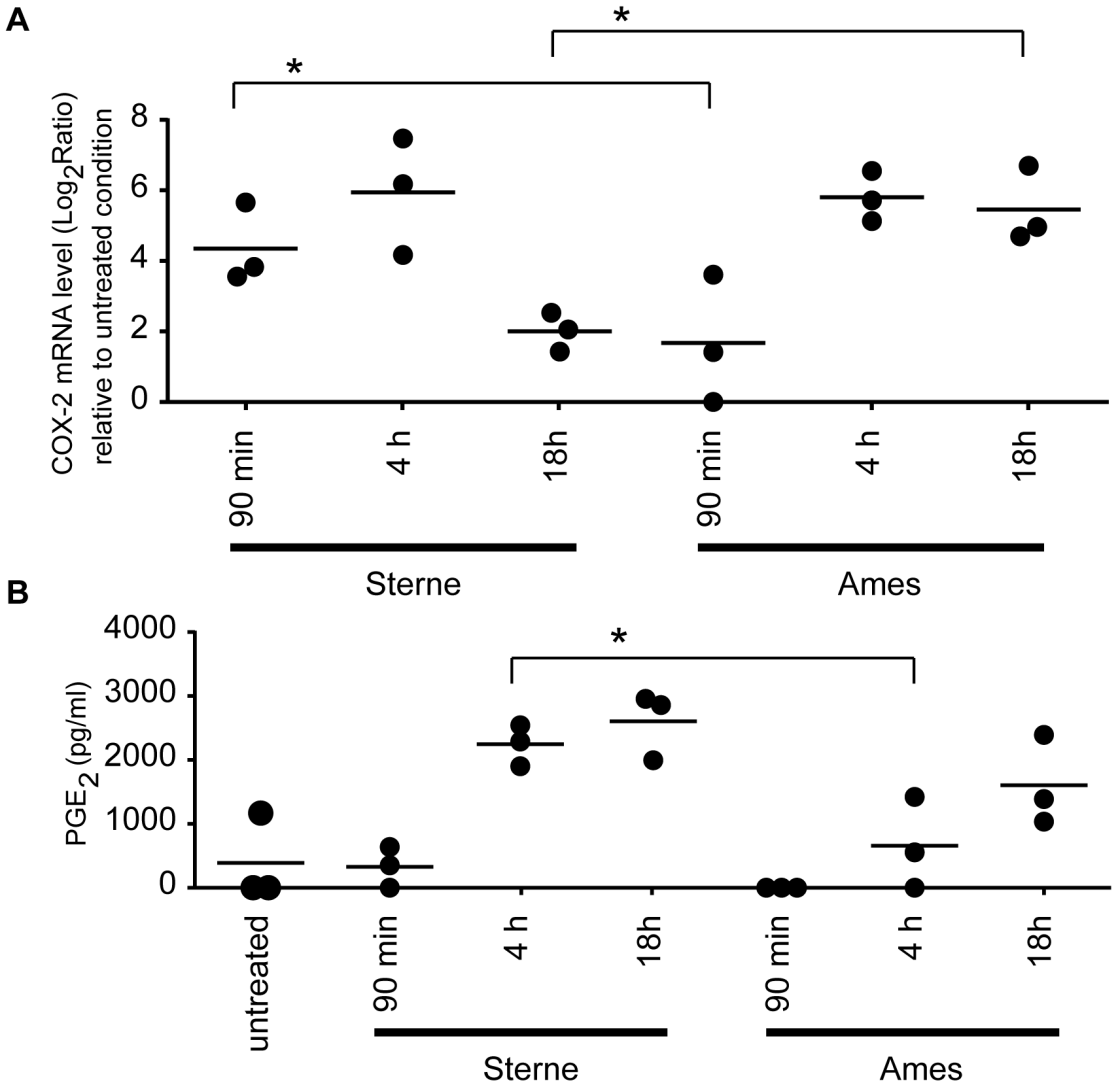
Time-dependent kinetic difference in the induction of COX-2 and PGE_2_ expression (A) AMs were infected with either Ames or Sterne spores for 90 min, 4 h or 18 h. Cells were lysed and the mRNA was purified and quantified by real time PCR. Fold expression was calculated by normalizing to time 0. (B) AMs were infected with Ames or Sterne spores at an MOI of 10. The amount of PGE_2_ was quantified by ELISA. Data shown in (A) and (B) are representative of n = 3 experiments. Scatter plots are presented as mean ± Standard deviation. P-value is calculated using a paired two tailed Student’s t-test. *represent p-value <0.05.

AMs initiate bactericidal activities once they phagocytose *B. anthracis* spores. Activated macrophages migrate to the draining lymph nodes to present antigens and assist in the activation of the adaptive immune response [19]. Homing of macrophages to draining lymph nodes is mediated by the proinflammatory chemokine receptor, CCR7, which can be up-regulated by PGE_2_ [19], [24]. PGE_2_ is derived from prostaglandin H_2_ which in turn is generated from arachadonic acid by the enzymatic action of COX-2 [25]–[27]. Furthermore, activated macrophages also release CCL5 and CCL20 that recruit immature DC and T cells, respectively, to the inflammatory site [28]–[30]. Our gene expression data showed increased levels of CCL5, CCR7 and COX-2 in Sterne-infected AMs than Ames-infected AMs (Figure S1). We, therefore, decided to further examine the expression pattern of COX-2 and PGE_2_ in AMs infected with Ames or Sterne spores. The COX-2 mRNA expression measured by real-time PCR confirmed the microarray analysis data (Figure 6A and Figure S1). After infecting AMs with Sterne spores, COX-2 mRNA was rapidly induced by 90 minutes. It continued to rise by ∼32 fold and peaked at 4 h post infection. However, COX-2 expression was attenuated by 18 h post infection. On the contrary, COX-2 mRNA expression was much reduced at 90 minutes following Ames spore infection, but continued to rise till 4 h and its expression was sustained up to 18 h post-infection. A statistically significant difference (p<0.05) was observed in COX-2 expression between Sterne and Ames at 18 hrs post-infection.

Prior studies have reported that COX-2 expression by macrophages leads to PGE_2_ production that furthers the progression of acute inflammation [31]. In fact, an increase in PGE_2_ production was observed in Sterne infected AMs over the 18 h time course (Figure 6B). Despite the fact that COX-2 mRNA expression in Sterne infected AMs is attenuated by 18 h, the amount of PGE_2_ detected is sustained. A statistically significant difference (p<0.05) in PGE_2_ production was observed between Sterne and Ames at 4 h post-infection. Furthermore, the time-dependent kinetics of PGE_2_ production in Ames infected AMs correlates to that of the COX-2 mRNA expression (Figure 6A). These studies suggest that modulation of the COX-2 gene expression and PGE_2_ lipid production may represent a strategy adopted by the host to effectively recruit macrophages to the site of infection after *B. anthracis* invasion.

### Anti-apoptotic and Pro-apoptotic Genes were Preferentially Up-regulated in Sterne and Ames Infected AMs, respectively

*B. anthracis* LF cleaves MEK1 and impairs the induction of p38 dependent anti-apoptotic genes. As a result, BMDMs from C57BL/6 undergo programmed cell death after exposure to LF [32]. Our microarray data identified 24 anti-apoptotic genes that are preferentially up-regulated in Sterne infected AMs (Table S2). Of these 24 anti-apoptotic genes, 11 and 13 of these genes were expressed at early (up to 120 mins post-infection) and late (from 120 to 360 mins post-infection) stages, respectively. The expressions of these anti-apoptotic genes may contribute to the survival of AMs after Sterne infections. In these studies, we measured the MEK1 cleavage at either very early (30 mins) or late (360 mins) time point post-infection. Although we have not measured the MEK1 cleavage pattern at the intermediate time points, we speculate that up to 120 mins the amount of LF being produced is not sufficient to degrade MEK1. As a result, the MAPK signaling cascade is intact; p38 dependent transcription factors and NF-κB are able to up-regulate the anti-apoptotic genes that are expressed at early and late time points [33]. At 6 h post Ames or Sterne infection MEK1 degradation was observed. As a result, p38 can no longer be phosphorylated (Figure 2A). Promoter analysis reveals multiple putative p38 dependent transcription factor binding sites in these 13 anti-apoptotic genes that are expressed at later stage (Figure S2) [34]. It is unclear how 13 anti-apoptotic genes are regulated in the absence of p38 activity at later stage of infection. The requirement of p38 dependent transcription factors in mediating the expression of these genes, therefore, needs to be empirically determined. Strikingly, instead of up-regulating anti-apoptotic genes, 14 pro-apoptotic genes are induced in Ames infected AMs (Figure 4). The distinct differences in apoptosis regulation further delineate the differences in pathogenicity between Ames and Sterne strains.

**Figure 7:**
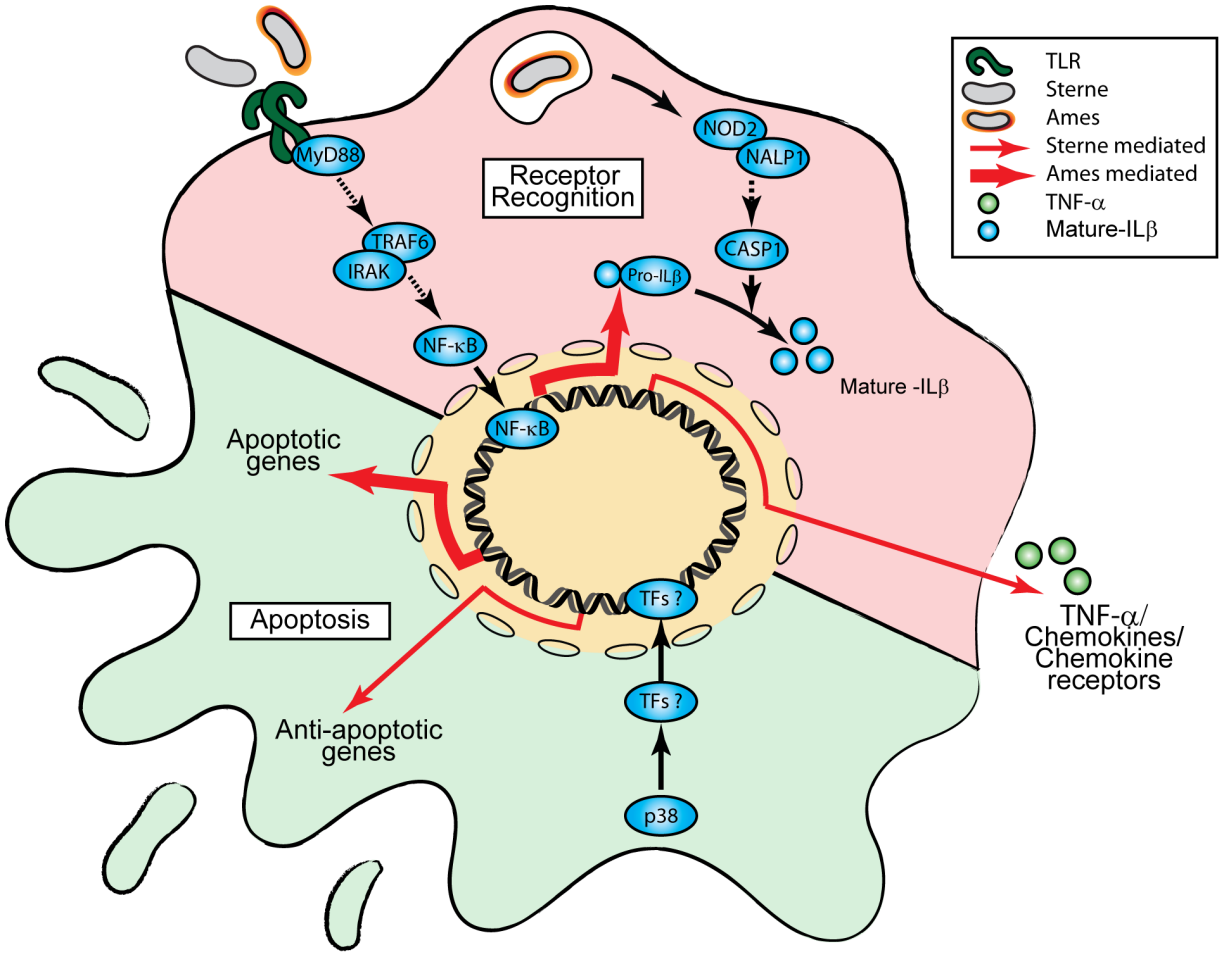
A Schematic diagram of observed differential modulation of host responses to Ames and Sterne spores of *B. anthracis*. The diagram is based on the observed differences in the cytokine/chemokine and host responses in AMs infected with either Ames or Sterne spores. TFs: Transcription factors.

In summary, this study presents evidence that infection of AMs with either Sterne or Ames spores results in time-dependent quantitative differences in gene expression and protein production of certain cytokines, chemokines and host factors. In particular, TNF-α or IL-1β protein secretions were up-regulated in Sterne or Ames infected AMs, respectively. Preferential expression of either TNF-α or IL-1β suggests different signal transduction pathways downstream of TLR and NOD2 are activated. The ability of *B. anthracis* to activate multiple innate immune receptors may govern the downstream pro-inflammatory cytokine production. Furthermore, differential expression of apoptotic versus anti-apoptotic genes may contribute to the survival of AMs in response to *B. anthracis* infections. We observed that AMs infected with Ames spores up-regulated apoptotic genes whereas AMs infected with Sterne spores up-regulated anti-apoptotic genes (Figure 7). These studies also suggest that the observed differences in host response are probably not due to the delay in the uptake of the Ames spores as different gene sets are modulated following Ames or Sterne infection. Lastly, the observed time-dependent kinetic differences in the production of COX-2 and PGE_2_ may lead to a more robust immune response in AMs infected with Sterne spores and a slower onset of immune response in Ames-infected AM. Together, these studies provide insight into the differential host cellular mechanisms employed by the virulent Ames or vaccine Sterne strain of *B. anthracis*.

## Supporting Information

Figure S1 **Time-dependent induced mRNA expression of CCL5, CCL3, COX-2 and CCR7 following infection of AMs with either Sterne or Ames spores.** AMs obtained from five rhesus macaque donors were infected with Ames or Sterne spores at an MOI of 10 for indicated time points. Total mRNAs were purified and hybridized to rhesus macaque cDNA microarrays. Data shown is representative of n = 5 experiments.
(TIF)

Figure S2 **Transcription factors that are predicted to regulate the expression of anti-apoptotic genes.** Boxes highlighted in yellow are transcription factors that can potentially be regulated by p38.
(TIF)

Table S1 **Hierarchical clustering of the microarray data.** Tab “Data” is the underlying data for Figure 4. Explanation of the column headings in Tab “Data” can be found in the tab named “Description”.
(XLSX)

Table S2 **Statistically significant overrepresentation of selected functional classes and protein families based upon gene ontology (GO) analysis.** Tab “GO ANALYSIS” is the underlying data for Figure 5. Explanations of the column headings in Tab “GO ANALYSIS” are: Subset: the subset that the GO term belongs to base on Figure 4; GO Term: the description of the biological processes; P-value: the P-value of the overlap; Gene ID: Entrez gene ID that belongs to the GO Term; Gene Symbol: the gene symbol of the corresponding gene ID.
(XLSX)
