# Supplementary figures and images for: Alveolar Macrophages Infected with Ames or Sterne Strain of Bacillus anthracis Elicit Differential Molecular Expression Patterns
Source: PLoS One. 2014 Feb 7;9(2):e87201. doi: 10.1371/journal.pone.0087201 (PMC3917846; doi:10.1371/journal.pone.0087201)

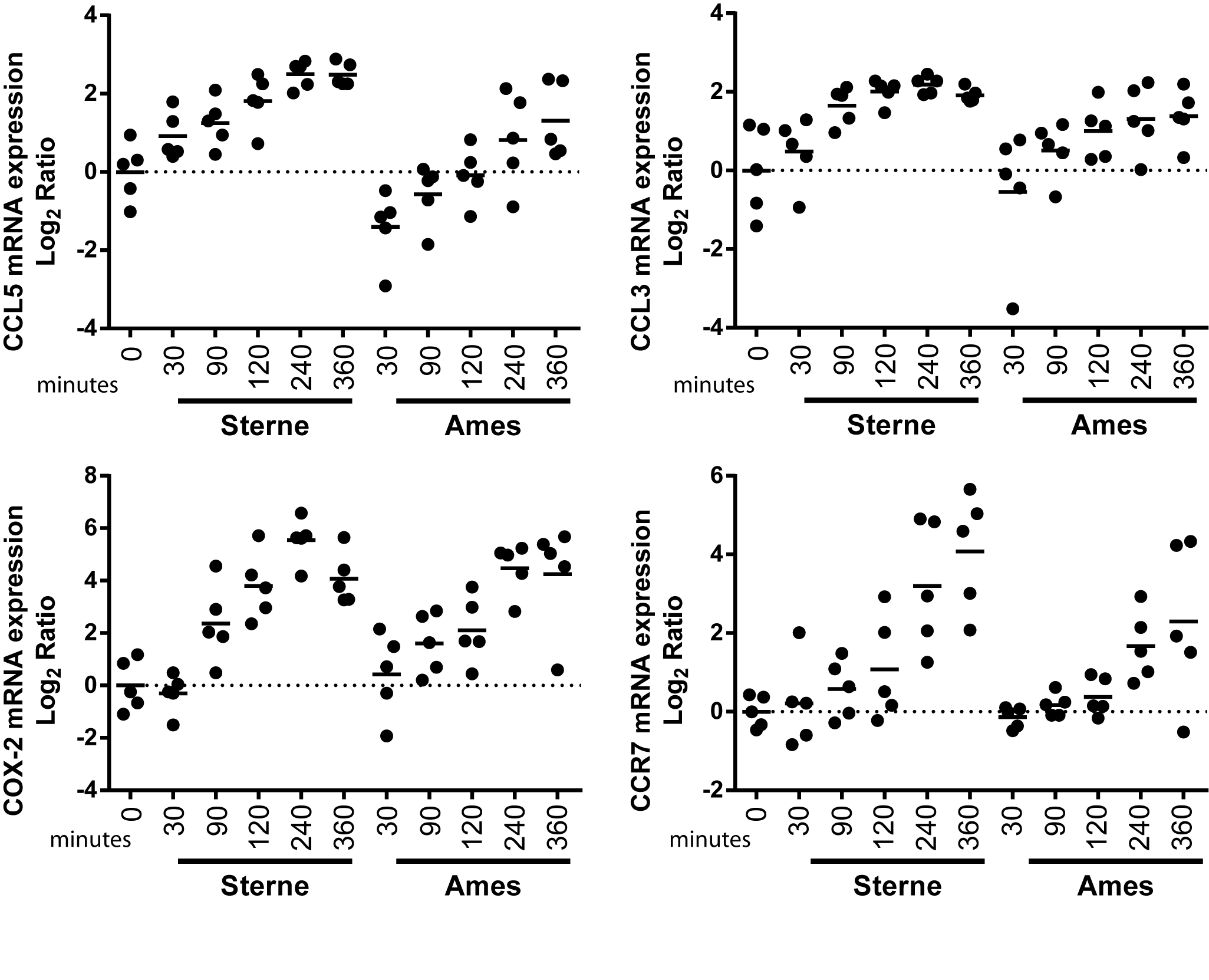

Supplement: Figure S1 — Time-dependent induced mRNA expression of CCL5, CCL3, COX-2 and CCR7 following infection of AMs with either Sterne or Ames spores. AMs obtained from five rhesus macaque donors were infected with Ames or Sterne spores at an MOI of 10 for indicated time points. Total mRNAs were purified and hybridized to rhesus macaque cDNA microarrays. Data shown is representative of n = 5 experiments. (TIF) [file pone.0087201.s001.tif]

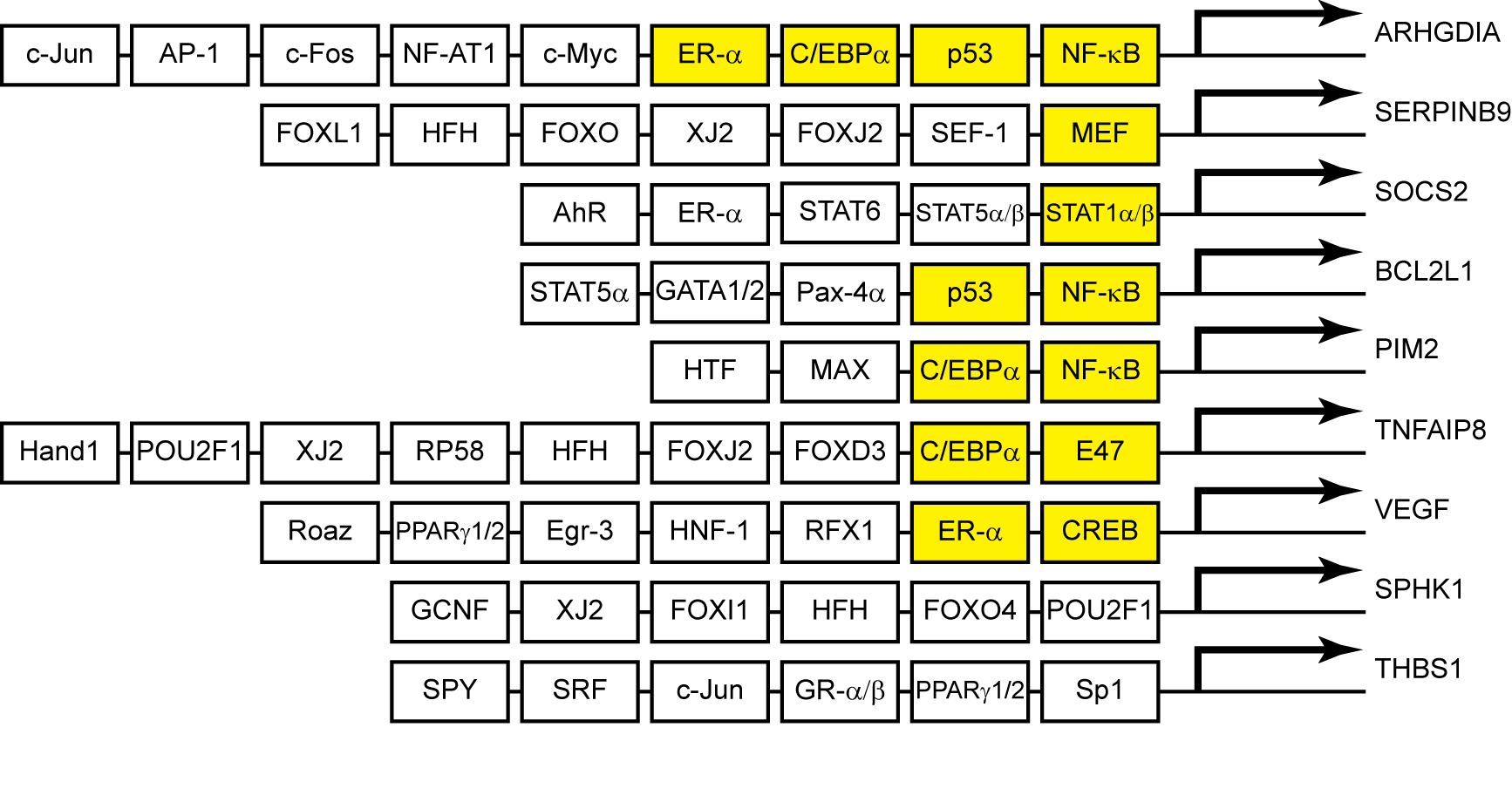

Supplement: Figure S2 — Transcription factors that are predicted to regulate the expression of anti-apoptotic genes. Boxes highlighted in yellow are transcription factors that can potentially be regulated by p38. (TIF) [file pone.0087201.s002.tif]
